# *S100B* gene polymorphisms are associated with the S100B level and Alzheimer’s disease risk by altering the miRNA binding capacity

**DOI:** 10.18632/aging.203005

**Published:** 2021-05-12

**Authors:** Jiafeng Wang, Yulan Zhou, Yixia Yang, Xiang Gao, Zhibin Liu, Guanhao Hong, Lifen Yao, Jingwen Yin, Xuefeng Gu, Keshen Li

**Affiliations:** 1 Stem Cell Research and Cellular Therapy Center, Affiliated Hospital of Guangdong Medical University, Zhanjiang, Guangdong, China; 2 Clinical Medicine Research Center, Affiliated Hospital of Guangdong Medical University, Zhanjiang, Guangdong, China; 3 Department of Neurology, The First Affiliated Hospital, Harbin Medical University, Harbin, Heilongjiang, China; 4 Department of Psychiatry, Affiliated Hospital of Guangdong Medical University, Zhanjiang, China; 5 Shanghai Key Laboratory of Molecular Imaging, Collaborative Research Center, Shanghai University of Medicine & Health Sciences, Shanghai, China; 6 Department of Neurology and Stroke Center, The First Affiliated Hospital of Jinan University, Guangzhou, China; 7 Clinical Neuroscience Institute of Jinan University, Guangzhou, China; 8 Guangdong Key Laboratory of Age-Related Cardiac and Cerebral Diseases, Affiliated Hospital of Guangdong Medical University, Zhanjiang, Guangdong, China

**Keywords:** *S100B*, SNP, miRNA, regulation, Alzheimer’s disease

## Abstract

To examine the role of *S100B* in genetic susceptibility to Alzheimer’s disease (AD), we conducted a case-control study to analyze four polymorphism loci (rs2839364, rs1051169, rs2300403, and rs9722) of the *S100B* gene and AD risk. We found an independent increased risk of AD in *ApoE* ε4(-) subjects carrying the rs9722 AA-genotype (OR = 2.622, 95% CI = 1.399–4.915, *P* = 0.003). Further investigation revealed the serum S100B levels to be lower in rs9722 GG carriers than in rs9722 AA carriers (*P* = 0.003). We identified three miRNAs (miR-340-3p, miR-593-3p, miR-6827-3p) in which the seed match region covered locus rs9722. Luciferase assays indicated that the rs9722 G allele has a higher binding affinity to miR-6827-3p than the rs9722 A allele, leading to a significantly decreased fluorescence intensity. Subsequent western blot analysis showed that the S100B protein level of SH-SY5Y cells, which carry the rs9722 G allele, decreased significantly following miR-6827-3p stimulation (*P* = 0.009). The present study suggests that the rs9722 polymorphism may upregulate the expression of *S100B* by altering the miRNA binding capacity and may thus increase the AD risk. This finding would be of great help for the early diagnosis of AD.

## INTRODUCTION

Alzheimer’s disease (AD) is the most common neurodegenerative illness and the leading cause of dementia in elderly people. Inflammation, insoluble protein deposition and neuronal cell loss are important features in the brains of AD patients [1]. At present, it is widely believed that maladaptive astrocytic activation constitutes a pathogenic mechanism of AD [2]. S100B, expressed primarily by astrocytes, is associated with the neuropathological hallmarks of AD; S100B also causes neuroinflammation and neurotoxicity [3, 4].

S100B belongs to the large family of S100 proteins, which are EF-hand calcium-binding proteins that exert both intracellular and extracellular effects on a variety of cellular processes [5–7]. Intracellularly, S100B modulates microtubule assembly and regulates the cell cycle. Extracellularly, the action of S100B is strongly dependent on its concentration [8]. Extracellular S100B shows a neuroprotective effect at the nanomolar level. However, at micromolar levels, extracellular S100B can stimulate the receptor for advanced glycation end products (RAGE) in neurons, leading to an overproduction of reactive oxygen species and, ultimately, resulting in apoptosis [9, 10] and upregulation of several proinflammatory cytokines [11]. At high concentrations, S100B also upregulates nitric oxide (NO) synthase, and stimulates NO release by microglia through synergy with bacterial endotoxin and IFN-γ, thereby participating in microglia activation [12].

In addition to its functional relevance, the human *S100B* gene is located on chromosome 21q22.3 [13] within or near risk regions for familial late-onset AD. To date, several single nucleotide polymorphisms (SNPs) of the *S100B* gene—particularly rs9722 and rs1051169—have been shown to affect the S100B protein level. Recently, Hohoff suggested the important role of *S100B* polymorphisms in S100B serum concentrations and *S100B* mRNA expression [14]. Moreover, *S100B* SNPs are significantly associated with several neuropathological and psychiatric disorders, such as Parkinson's disease [15, 16], depressive disorder [17], schizophrenia [18, 19], stroke [20] and dementia [21]. However, very few investigations of connecting *S100B* polymorphisms with AD risk have been performed, especially in the Chinese Han population. Thus, herein, we conducted a case-control study in that population to examine the potential role of *S100B* polymorphisms in AD risk.

An increasing number of experimental studies and clinical examinations have reported elevated levels of S100B in the brains or cerebrospinal fluid of AD patients [22–25], which implies that *S100B* is closely tied to the pathogenesis of AD. Additional supporting evidence comes from a recent study that used double transgenic mice that overexpressed *S100B* and carried the *APP* mutation (Tg2576/*S100B*), which promotes brain inflammation characterized as astrogliosis and microgliosis and enhances Aβ generation [3]. Further, many previous animal studies have demonstrated that *S100B* dysregulation can alter Aβ deposits, plaques, and gliosis [3, 26, 27]. Owing to such evidence, *S100B* may act as an unconventional cytokine, playing a role in the pathophysiology of AD; *S100B* is therefore a plausible biological candidate as a susceptibility gene for AD.

MicroRNAs (miRNAs) are a class of single-stranded RNA molecules responsible for post-transcriptional gene silencing, usually by binding to the 3′ untranslated region (3′-UTR) of target genes that play critical roles in the regulation of target gene expression [28]. Recent studies have found that approximately 70% of experimentally detectable miRNAs are expressed in the brain, and some studies suggest that miRNAs are intimately involved in synaptic function and specific signals during memory formation. Increasing evidence implies the possible involvement of miRNAs in AD [29–32]. Using genome-wide profiling, Chang (2017) found five differently expressed genes (DEGs) regulated by four differently expressed miRNAs (DEmiRNAs) in AD. Swarbrick (2019) reported on 10 miRNAs that could be deregulated early in the peripheral blood of Alzheimer’s patients, nearly 20 years before the onset of clinical symptoms [33]. These miRNAs could serve as specific AD biomarkers, which may provide the basis for a novel, effective diagnostic approach and new targets for pharmaceutical development.

Limited studies have focused on the miRNAs that regulate the S100B level. Chen (2018) showed that *S100B* genes are the targets of miR-330-3p, and lncRNA X-inactive specific transcript (XIST) promotes *S100B* expression by harboring complementary binding sites with miR-330-3p, eventually preventing cardiac hypertrophy [34]. Using a cerebral palsy rat model, Wen (2020) discovered that the overexpression of miR-135b can downregulate *S100B*, which helps to inactivate the signal transducer and activator of transcription-3 (STAT3) pathway, and promotes neural stem cell (NSC) differentiation and proliferation but inhibits NSC apoptosis [35]. Recently, Chen (2020) reported that plasma miR-340-3p and S100B levels differ significantly among various rs9722 genotypes and that the *S100B* rs9722 locus SNP is associated with the risk of chronic heart failure. This finding signals that miR-340-3p may play different roles in regulating the S100B level among different *S100B* rs9722 genotypes [36].

In this study, we analyzed the plasma S100B levels in AD patients with different *S100B* genotypes. To explore in detail how SNPs are related to AD, we determined whether the SNPs in the 3′-UTR of the *S100B* gene could affect the S100B level by altering the combination of miRNAs and *S100B* mRNAs.

## RESULTS

### Distributions of the genotype and allele frequencies of S100B polymorphisms

**Figure 1:**
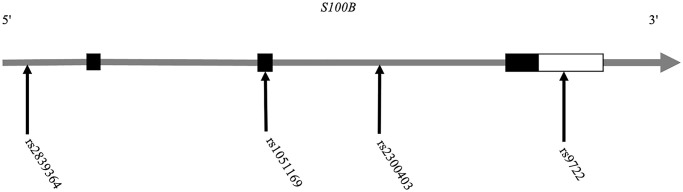
Position distribution of the SNPs in the *S100B* gene.

In total, we recruited 280 AD patients and 400 healthy control individuals. Using the SNaPshot method, we chose and genotyped the four most reported phenotypically relevant SNPs of the *S100B* gene (rs2839364 in the promoter region, rs1051169 in the second exon, rs2300403 in the second intron and rs9722 in the 3′UTR of the *S100B* gene) (Figure 1).

Table 1 summarizes the genotypes and allele frequencies of the four polymorphisms. We did not observe deviations from Hardy–Weinberg equilibrium (HWE) for the candidate polymorphisms in the control group. We noted significant differences in the genotype frequencies of rs9722 between the AD patients and the controls (OR = 2.029, 95% CI = 1.243–3.311, *P* = 0.005). Likewise, we observed significant differences in the allele distributions of rs9722 between the AD patients and the controls (OR = 1.342, 95% CI = 1.085–1.661, *P* = 0.008). The mutant alleles (rs9722 T) were more frequent in the AD population than in the control population. However, no significant differences in the allele distributions of rs1051169 between the AD patients and the controls was observed. In addition, neither the genotype nor allele frequency displayed significant differences between the AD patients and the controls for the rs2839364 and rs2300403 polymorphisms.

**Table 1:** Genotype and allele frequency distributions of the *S100B* SNPs in the AD patients and healthy controls.

**SNPs**	**genotype**	**AD**	**control**	***p***	**OR (95% CI)**	**allele**	**AD**	**control**	***p***	**OR (95% CI)**
rs2839364	CC	283	291							
	CT	91	90	0.865	1.040 (0.745–1.451)	C	657	672		
	TT	25	19	0.352	1.353 (0.729–2.513)	T	141	128	0.385	1.126 (0.867–1.464)
ApoE ɛ4(−)	CC	175	235							
	CT	59	77	0.920	1.029 (0.695–1.522)	C	409	547		
	TT	20	13	0.067	2.066 (1.055–4.273)	T	99	103	0.119	1.285 (0.949–1.742)
ApoE ɛ4(+)	CC	108	56							
	CT	32	13	0.593	1.277 (0.621–2.625)	C	248	125		
	TT	5	6	0.200	0.432 (0.126–1.477)	T	42	25	0.577	0.847 (0.494–1.453)
rs1051169	GG	166	179							
	GC	158	168	0.938	1.021 (0.754–1.381)	G	490	526		
	CC	75	53	0.049	1.526 (1.012–2.300)	C	313	274	0.063	1.215 (0.992–1.489)
ApoE ɛ4(−)	GG	97	144							
	GC	104	139	0.581	1.111 (0.774–1.595)	G	298	427		
	CC	42	42	0.126	1.485 (0.901–2.446)	C	188	223	0.135	1.208 (0.946–1.542)
ApoE ɛ4(+)	GG	69	35							
	GC	54	29	0.878	0.945 (0.515–1.734)	G	192	99		
	CC	33	11	0.336	1.522 (0.688–3.368)	C	126	51	0.262	1.274 (0.849–1.911)
rs2300403	AA	189	194							
	AG	156	168	0.764	0.953 (0.709–1.282)	A	534	556		
	GG	52	37	0.128	1.443 (0.905–2.299)	G	260	242	0.306	1.119 (0.905–1.383)
ApoE ɛ4(−)	AA	115	157							
	AG	98	144	0.720	0.929 (0.654–0.321)	A	328	458		
	GG	30	24	0.099	1.706 (0.948–3.077)	G	158	192	0.299	1.149 (0.891–1.481)
ApoE ɛ4(+)	AA	74	37							
	AG	58	24	0.639	1.208 (0.651–2.242)	A	206	98		
	GG	22	13	0.687	0.846 (0.384–1.866)	G	102	50	0.916	0.971 (0.641–1.471)
rs9722	GG	182	209							
	GA	165	161	0.294	1.177 (0.877–1.580)	G	529	579		
	AA	53	30	**0.005**	**2.029 (1.243**–**3.311)**	A	271	221	**0.008**	**1.342 (1.085**–**1.661)**
ApoE ɛ4(−)	GG	111	169							
	GA	94	138	0.857	1.037 (0.727–1.479)	G	316	468		
	AA	31	18	**0.003**	**2.622 (1.399**–**4.915)**	A	156	166	**0.013**	**1.393 (1.072**–**1.808)**
ApoE ɛ4(+)	GG	71	40							
	GA	71	41	0.929	0.976 (0.565–1.684)	G	213	121		
	AA	22	12	0.937	1.033 (0.463–2.306)	A	93	65	0.320	0.813 (0.552–1.198)

*P* values under 0.0167 were indicated in bold font.

### Stratified analyses

We also carried out stratified analyses according to the *ApoE* ε4 allele. An increased risk of AD was more evident among *ApoE* ε4(-) subjects carrying the rs9722 AA-genotype (OR = 2.622, 95% CI = 1.399–4.915, *P* = 0.003). Likewise, the allele distributions of rs9722 between the AD patients and the controls was significantly different in the *ApoE* ε4(-) subjects (OR = 1.393, 95% CI = 1.072–1.808, *P* = 0.013). Nevertheless, we did not find any association between the different genotypes of rs1051169 and AD risk after performing stratified analyses based on the *ApoE* ε4 allele. In addition, we did not observe more apparent associations between rs2839364 or rs2300403 and AD risk among the subgroups via the *ApoE* ε4 allele (Table 1).

### Haplotype association analyses

We performed haplotype association analyses of the four polymorphic loci. We identified and evaluated the haplotypes of the four SNPs. We found four main haplotypes; the C-G-A-G haplotype (in the order of rs2839364, rs1051169, rs2300403 and rs9722) was the most prevalent in both the AD population and the controls. Assigning the most common haplotype C-G-A-G as the reference, the haplotype C-C-G-A showed significant differences between the AD patients and the controls (OR = 1.605, 95% CI = 1.189–2.165, *P* = 0.002). We did not find any more significant differences between the AD patients and the controls for the other two main haplotypes (Table 2).

**Table 2:** Haplotype frequencies of the *S100B* SNPs in the AD patients and controls.

**Haplotypes**	**Control (freq)**	**AD (freq)**	***P***	**OR 95% CI**
C-G-A-G	485.31 (0.607)	421.79 (0.527)	1	−
C-C-A-G	121.94 (0.152)	125.88 (0.157)	0.251	1.188 (0.896–1.1.572)
C-C-G-A	91.02 (0.114)	127.36 (0.159)	**0.002**	1.605 (1.189–2.165)
C-G-G-A	89.14 (0.111)	75.31 (0.094)	0.865	0.968 (0.693–1.351)

Haplotypes consisted of S100B SNPs rs2839364, rs1051169, rs2300403 and rs9722.

### Serum S100B levels analyses

**Figure 2:**
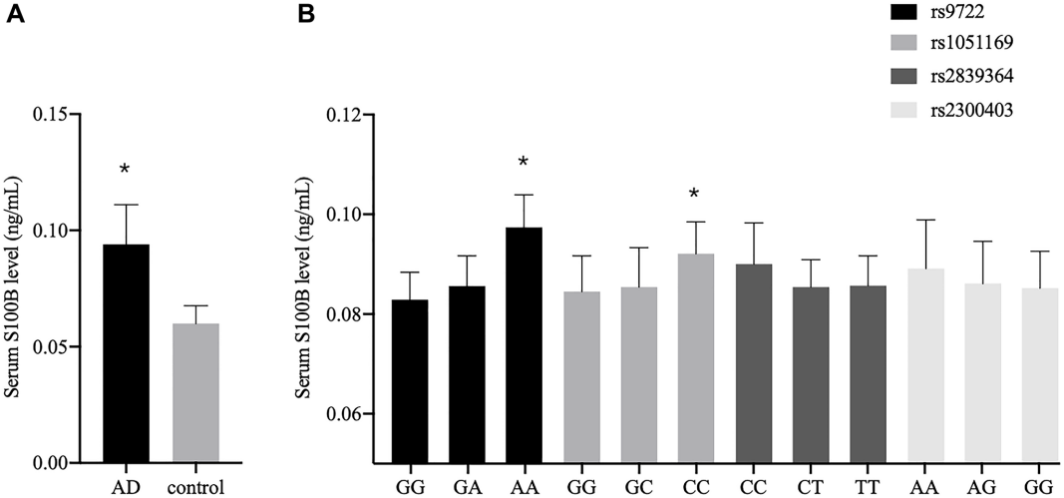
**Serum S100B levels among different groups.** (**A**) Serum S100B levels between the AD patients and the controls. (**B**) Serum S100B levels among different genotypes in the AD patients (^*^P < 0.05).

Since AD is closely linked to the *in vivo* S100B level, we compared the serum S100B levels in the AD patients and the healthy controls. The data revealed the average serum S100B level in AD patients to be significantly higher than that in the controls (*P* = 0.036) (Figure 2A). We also investigated the association between the *S100B* polymorphisms and the serum S100B levels. As shown in Figure 2B, the serum S100B levels were significantly upregulated in the rs9722 AA genotype compared to the rs9722 GG genotype in the AD patients (*P* = 0.003). Similarly, the serum S100B levels were significantly upregulated in the rs1051169 CC genotype compared to the rs1051169 GG genotype in the AD patients (*P* < 0.001). We did not observe any significant differences between patients carrying the mutated genotypes and wild-type genotypes of the rs2839364 and rs2300403 polymorphisms.

### Luciferase assay detection

**Figure 3:**
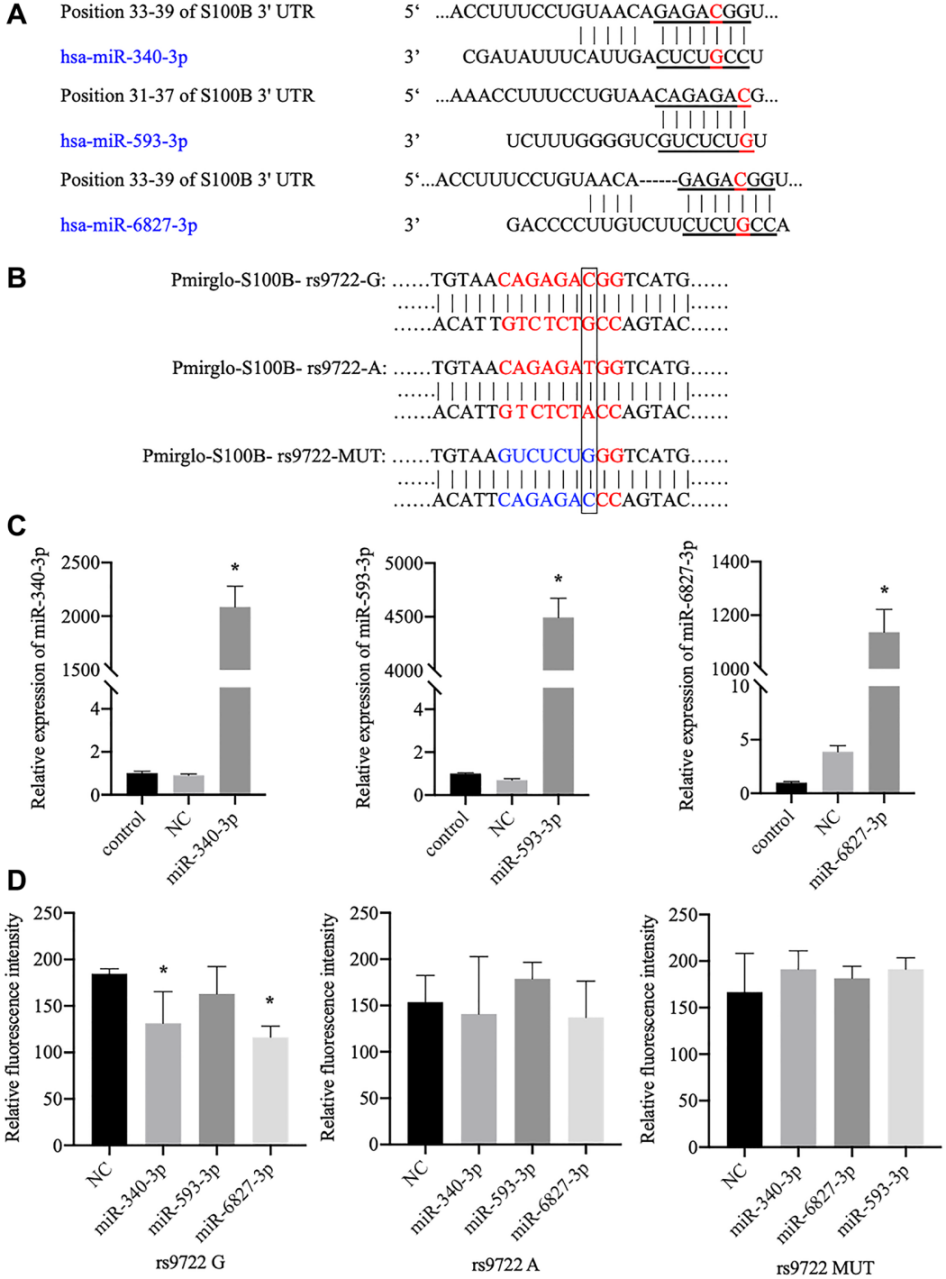
**Verification of the interaction between candidate miRNAs and the 3′-UTR of the *S100B* gene, which contains the rs9722 loci, using the luciferase assay.** (**A**) The candidate miRNAs that can bind to the rs9722 locus in the 3′-UTR of the *S100B* gene. The red letters show the rs9722 locus. (**B**) Plasmids containing different genotypes and artificial mutations. The black frame indicates the rs9722 locus. (**C**) Detection of miRNA levels in the 293T cells after transfection. (**D**) Fluorescence intensity after transfection of the miRNAs and the plasmids containing the different rs9722 alleles or mutations (^*^P < 0.05).

After using online software prediction, we identified three miRNAs (miR-340-3p, miR-593-3p, and miR-6827-3p) in which the seed match region covered locus rs9722 (Figure 3A). A subsequent luciferase assay indicated that miR-340-3p and miR-6827-3p stimulation could significantly reduce the fluorescence intensity of 293T cells that contained the rs9722 G allele (rather than the rs9722 A/MUT allele) (Figure 3B). We did not observe any significant decrease in fluorescence intensity following stimulation by miR-593 (Figure 3C, 3D).

### Western blot detection

**Figure 4:**
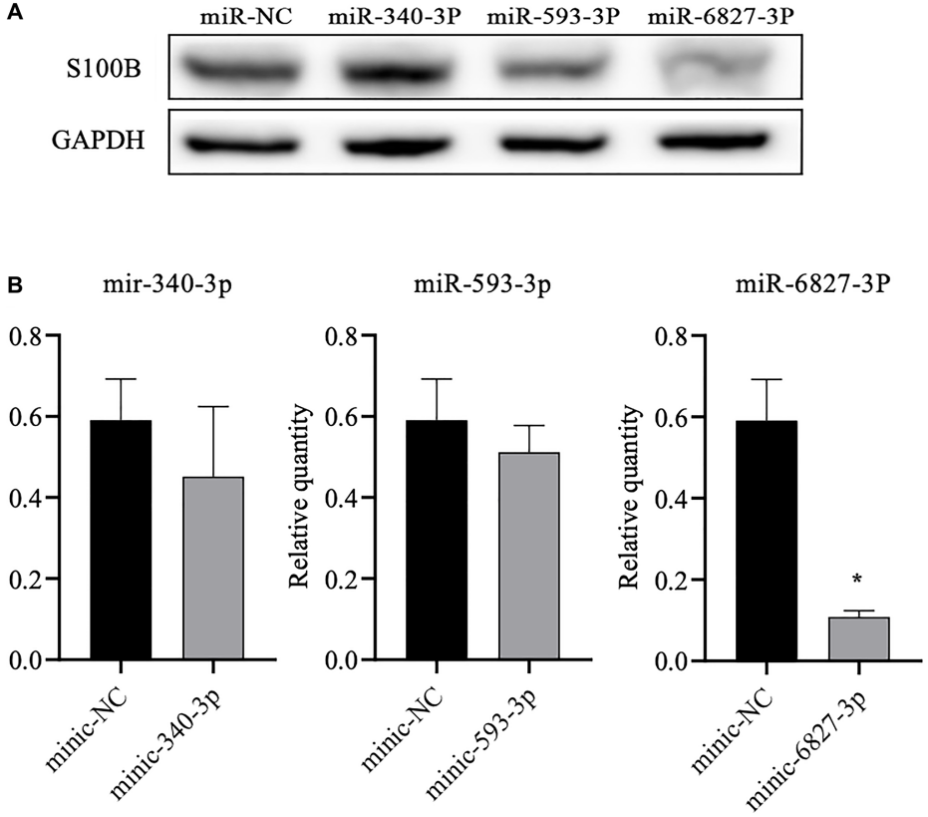
**Western blot analysis of the S100B levels in SH-SY5Y cells after transfection of the three different miRNAs.** (**A**) We detected the S100B protein level using western blot analysis. (**B**) Densitometry analysis to determine the ratio of S100B to GAPDH. All data are the average of three measurements (^*^P < 0.05).

The subsequent western blot data showed that miR-6827-3p could significantly reduce the S100B level of the SH-SY5Y cells containing the rs9722 G allele (*P* = 0.009). We did not observe a significant difference, although the S100B level decreased following stimulation by miR-340-3p. Likewise, we did not detect a significant difference in the S100B protein level following stimulation by miR-593-3p (Figure 4).

## DISCUSSION

Some studies have verified that S100B may contribute to the pathogenesis of AD, revealing that S100B persists in the extracellular space and upregulates chronically activate RAGE in astrocytes or microglia, thereby amplifying the inflammatory response [37–39]. Extracellular S100B promotes RAGE-dependent hyperphosphorylation of tau protein and the development of neurofibrillary tangles (NFTs) [40]. Moreover, a large number of clinical studies have demonstrated that the S100B levels are elevated in the CSF and/or serum of patients with various neuropsychiatric diseases, including schizophrenia [41, 42], bipolar disorder [43], ischemic stroke [20], multiple sclerosis [44] and AD [22, 24]. We found the serum S100B level of AD patients to be significantly higher than that of healthy controls (Figure 2A). Increasing S100B levels in the brain can accelerate cerebral amyloidosis, possibly by promoting the cleavage of APP to Aβ [4, 45]. Additionally, S100B may promote the conversion of diffuse, non-fibrillar Aβ deposits into neuritic Aβ plaques, thus exacerbating the progression of AD pathology [4].

In this case-control study, we found the *S100B* SNP loci rs9722 to be associated with AD risk. We also examined the association of the serum S100B level with respect to the corresponding *S100B* polymorphisms in AD patients. The data revealed that the AA genotype of the rs9722, as well as the CC genotype of the rs1051169 locus, are significantly more associated with higher serum S100B levels. This outcome confirmed the finding reported by Hohoff (2010) regarding the potential role of *S100B* SNPs in S100B serum concentrations in a healthy population [14]. In addition, the expression quantitative trait loci (eQTL) data of *S100B* in whole blood from the GTEx Portal showed that the serum S100B levels were significantly upregulated in the AA genotype compared to the GG genotype of the rs9722 locus. Likewise, the serum S100B levels were significantly upregulated in the CC genotype compared to the GG genotype of the rs1051169 locus (Supplementary Figure 1). This finding is in line with our results. The alleles of the two SNPs mentioned above, which were shown to influence *S100B* gene expression, became risk factors that were also indirectly confirmed by the haplotype analysis. Thus, the haplotype C-C-G-A contained both the rs1051169 C allele and the rs9722 A allele, indicating a significant association with AD risk (Table 2).

Lambert (2007) found that the *S100B* polymorphism rs2300403 was correlated with low cognitive performance and dementia in elderly people, thereby underlining the importance of *S100B* in genetic susceptibility to AD [21]. However, we did not detect any significant association of rs2300403 with AD in our study, probably because Lambert analyzed the *S100B* mRNA level, while we focused on the serum S100B protein level. Hence, we speculate that post-transcriptional regulation may play a role in the *S100B* translation process.

The SNP rs9722 is located in the 3′-UTR of *S100B* (Figure 1); 3′-UTRs typically contain important regulatory elements, such as U-rich motifs, AU-rich elements, or microRNA target sites, which modulate gene expression and may also apply to rs9722 [46]. We examined the microRNA binding sites in the 3′-UTR of the *S100B* gene and identified three miRNAs (miR-593-3p, miR-340-3p and miR-6827-3p) that bind precisely to the rs9722G allele (Figure 3A).

The subsequent luciferase assay data verified that miR-6827-3p can bind to the 3′-UTR of *S100B* mRNA (Figure 3D). In addition, the western blot assay signaled a significant decreasing S100B protein level in SH-SY5Y cells containing rs9722 G after stimulation by miR-6827-3p (Figure 4). Therefore, we believe that miR-6827-3p can specifically bind to the 3′-UTR of *S100B* mRNA in individuals carrying the rs9722 G allele, which results in the degradation of *S100B* mRNA and a subsequently lower S100B protein level. The rs9722 A allele reduced the stability of miR-6827-3p in binding to the *S100B* mRNA, thereby preventing the S100B protein from decreasing, which may explain why the rs9722 A allele seems to be a risk factor for AD.

We found that the GG genotype of the rs1051169 locus is also significantly related to the increased serum S100B level in AD patients. However, after stratified analyses based on the *ApoE* ε4 allele, we did not observe any significant genotypes/alleles frequencies differences between the AD patients and the controls of rs1051169 (Table 1). We speculate that the varying S100B levels between different rs1051169 genotypes may be due to a genetic linkage or other nongenetic influence factors.

Taking these pieces of evidence together, we can hypothesize that a possible intrinsic factor within the *S100B* gene may play a role in regulating *S100B* expression. Although S100B levels might be mediated by a possible feedback loop induced by the binding receptors of *S100B* (like RAGE), we observed indications that an intrinsic genetic factor could be partially involved in regulating *S100B* expression. The mechanism of AD is still unclear, but it may at least be partially explained by the increased *S100B* expression due to different *S100B* genotype carriers.

In addition, we observed an interesting phenomenon in which the distribution of the rs9722 genotype was quite different between our population and the German population. The genotype frequency of AA+AG in our healthy population was 47.8% (191/400), which is much higher than in the German population (18.4%, 36/160) reported by Hohoff [14]. Given that the distribution of the *S100B* gene frequencies might vary among different ethnic groups, this discrepancy suggests that studies need to be performed with a larger ethnically diverse population to clarify whether the association is limited to the Chinese Han population or whether it could also apply to other ethnic groups.

In sum, our study implies a significant association between the genotypes of *S100B* polymorphisms and increased incidence of AD in the Chinese Han population. Our findings highlight the rs9722 variant of *S100B* as an important risk factor for AD, which may act by regulating *S100B* expression. Further investigation revealed that different rs9722 genotypes may alter the combination of miR-6827-3p and *S100B* mRNA, which could subsequently decrease the S100B protein level. This is the first study to indicate an association between polymorphisms of the *S100B* gene and AD in an Asian cohort. In addition, our study signals the epigenetic regulation of *S100B* expression during the pathogenesis of AD. More research is necessary to substantiate these results and to shed light on the importance of interethnic differences affecting the outcomes of the complex nature of AD.

## MATERIALS AND METHODS

### Study subjects

We recruited all participants from the First Affiliated Hospital of Harbin Medical University in North China. The patient group consisted of 280 individuals (mean age at onset: 69.68 ± 8.27 years; mean age: 71.86 ± 9.07 years; 49.6% male). All patients met the criteria of the National Institute of Neurological and Communicative Disorders and Stroke as well as of the Alzheimer’s Disease and Related Disorders Association for probable AD. We excluded patients with a family history of dementia. The control group was composed of 400 healthy individuals (mean age: 74.21 ± 8.33 years; 51.5% male) recruited from individuals who underwent a regular health examination at the same hospital. The participants were confirmed to be healthy and neurologically normal using the Mini-Mental State Examination, the Revised Hasegawa Dementia Scale and general examinations. All participants were representative of the northern Chinese Han population living in North China, as defined by the geography of the Yellow River. Both groups were matched for geographic location, ethnicity, sex and age. We obtained informed consent either directly from the participants or their guardians. The Medical Ethical Committee of the Affiliated Hospital of Guangdong Medical University reviewed and approved of the study’s protocol.

### Sample collection and SNPs genotyping

We took a 5-mL venous whole blood sample from each participant for DNA extraction and SNP genotyping (including rs2839364, rs1051169, rs2300403 and rs9722). We isolated genomic DNA from venous blood samples using the Blood Genomic DNA Extraction Kit (Tiangen, China). We genotyped polymorphisms of the samples using the ABI PRISM SNaPshot method (Applied Biosystems, Foster, CA). The assay conditions followed the manufacturer’s protocols. We genotyped 10% of the samples in duplicate to verify the accuracy of the genotyping data.

### Cell lines acquisition and culture

We obtained the 293T and SH-SY5Y cell lines from ATCC (Manassas, VA, USA). We cultured cells in the DMEM, to which we added 10% FBS (Gibco, USA) at 37°C, and supplemented it with 5% CO_2_ using the Thermo HERAcell 150i incubator (Thermo, USA).

### Luciferase assay

All the miRNAs that could bind to the SNP loci in the 3′UTR of the *S100B* gene were predicted using the online software Targetscan (http://www.targetscan.org/vert_71/). Moreover, we employed the luciferase assay to evaluate the targeted binding relationship between the candidate miRNAs and the different SNP genotypes. We amplified the 3′UTR region sequences covering the SNP locus through specific PCR. We obtained the amplicons containing the different alleles from different homozygote individuals carrying different SNP genotypes. Then, we cloned the amplicons into the psi-CHECK2 vector (Promega, USA) and transfected them into 293T cells using lipofectamine3000 (Life Technology, USA). Additionally, we cotransferred the miRNA negative control and mimics (RiboBio, China) into 293T cells. After 48 hours of culture, we harvested the cells and subjected them to the luciferase activity tests of both firefly and renilla using the Dual-Luciferase Reporter Assay System (Promega, USA).

### Transfection and western blot

We constructed overexpressed plasmids containing different rs9722 genotypes and transfected them into SH-SY5Y cells using Lipofectamine™ 3000 Transfection Reagent (Invitrogen™, USA) according to the recommended protocol. We detected the S100B protein levels of SH-SY5Y cells with different genotypes using the western blot method following stimulation by the candidate miRNA mimics 48 hours later.

### RNA extraction and real-time quantitative PCR

We extracted total RNA using Trizol Regent (Invitrogen, USA) according to the recommended protocol. We detected the relative transcription levels of the gene/miRNAs through real-time quantitative PCR (qPCR) analysis. We adopted the 2^-ΔΔCT^ method and selected GAPDH and U6 as internal reference for genes and miRNAs, respectively. All the primers were summarized in the Supplementary Table 1.

### ELISA assay and S100B level detection

We used an enzyme immunoassay quantitative measurement, an ELISA Kit (KA0037, Abnova), for S100B to determine the serum S100B levels in 90 randomly selected venous blood samples. We also examined the eQTL data with the different genotypes of the four candidate SNPs in whole blood, and we obtained the eQTL data from the GTEx Portal (https://gtexportal.org/home/).

### Statistical analysis

We performed all analyses using SPSS version 19.0 (IBM, NY, USA). We counted and estimated the genotype and allele frequency distributions in the groups using the chi-square or Fisher’s exact test, and results were adjusted for multiple comparisons by Bonferroni correction. We assessed deviations of the genotype or allele frequency using HWE. We calculated the odds ratio (OR) and the 95% confidence interval (CI) to establish the correlation between the S100B genotype and AD.

## Supplementary Materials

Supplementary Figure 1

Supplementary Table 1
