# Supplementary material for: S100B gene polymorphisms are associated with the S100B level and Alzheimer’s disease risk by altering the miRNA binding capacity
Source: Aging (Albany NY). 2021 May 12;13(10):13954–67. doi: 10.18632/aging.203005 (PMC8202836; doi:10.18632/aging.203005)
Supplement: Supplementary Figure 1 [file aging-13-203005-s001.pdf]

SUPPLEMENTARY FIGURE

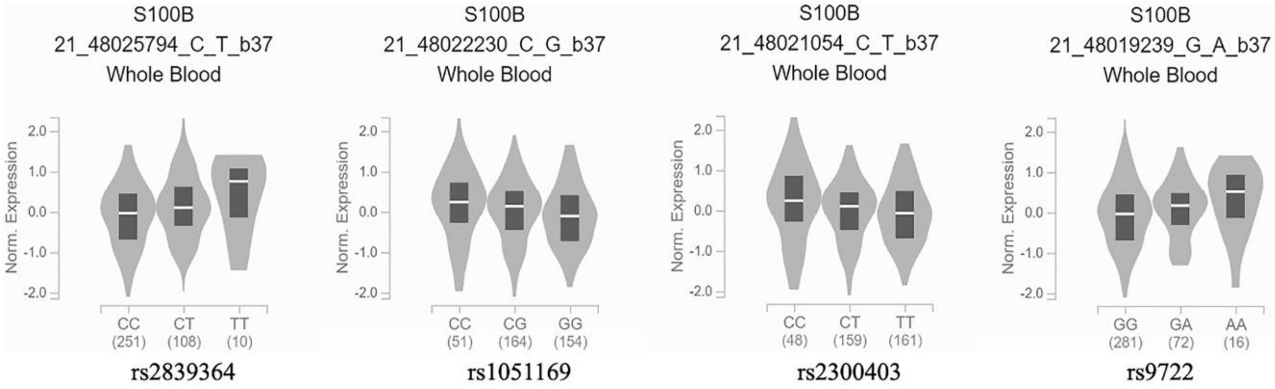

Supplementary Figure 1. eQTL data with different genotypes of the *S100B* SNPs in whole blood. The data come from the GTEx Portal (https://gtexportal.org/home/).
