# Supplementary material for: S100B gene polymorphisms are associated with the S100B level and Alzheimer’s disease risk by altering the miRNA binding capacity
Source: Aging (Albany NY). 2021 May 12;13(10):13954–67. doi: 10.18632/aging.203005 (PMC8202836; doi:10.18632/aging.203005)
Supplement: Supplementary Table 1 [file aging-13-203005-s002.pdf]

SUPPLEMENTARY TABLE

Supplementary Table 1. Real-time quantitative PCR primers used in this study.

primers	sequences	Amplicon length
U6-RT	GTCGTATCCAGTGCAGGGTCCGAGGTATTCGCACTGG ATACGACCAAATATGGAAC	94
U6-F	CTCGCTTCGGCAGCACA	
U6-R	AACGCTTCACGAATTTGCGT	
hsa-miR-340-3p-RT1	GTCGTATCCAGTGCAGGGTCCGAGGTATTCGCACTGG ATACGACCgctataaa	64
hsa-miR-340-3p-F1	ATGGTTCGTGGGTCCGTCTCAG	
Com R	GTGCAGGGTCCGAGGT	
hsa-miR-593-3p-RT1	GTCGTATCCAGTGCAGGGTCCGAGGTATTCGCACTGG ATACGACCagaaaccc	62
hsa-miR-593-3p-F1	ATGGTTCGTGGGTGTCTCTGC	
Com R	GTGCAGGGTCCGAGGT	
hsa-miR-6827-3p-RT1	GTCGTATCCAGTGCAGGGTCCGAGGTATTCGCACTGG ATACGACCctggggaa	65
hsa-miR-6827-3p-F1	ATGGTTCGTGGGACCGTCTCTTC	
Com R	GTGCAGGGTCCGAGGT	
